# miR-196a Is Able to Restore the Aggressive Phenotype of Annexin A1 Knock-Out in Pancreatic Cancer Cells by CRISPR/Cas9 Genome Editing

**DOI:** 10.3390/ijms19071967

**Published:** 2018-07-06

**Authors:** Raffaella Belvedere, Pasquale Saggese, Emanuela Pessolano, Domenico Memoli, Valentina Bizzarro, Francesca Rizzo, Luca Parente, Alessandro Weisz, Antonello Petrella

**Affiliations:** 1 Department of Pharmacy, University of Salerno, via Giovanni Paolo II 132, 84084 Fisciano (SA), Italy; rbelvedere@unisa.it (R.B.); epessolano@unisa.it (E.P.); vbizzarro@unisa.it (V.B.); lparente@unisa.it (L.P.); 2 Laboratory of Molecular Medicine and Genomics, Department of Medicine, Surgery and Dentistry ‘Scuola Medica Salernitana’, University of Salerno, via S. Allende, 1, 84081 Baronissi (SA), Italy; psaggese@unisa.it (P.S.); dmemoli@unisa.it (D.M.); frizzo@unisa.it (F.R.); aweisz@unisa.it (A.W.)

**Keywords:** annexin A1, pancreatic cancer, miR-196a-5p, EMT, biomarkers

## Abstract

Annexin A1 (ANXA1) is a Ca^2+^-binding protein that is involved in pancreatic cancer (PC) progression. It is able to mediate cytoskeletal organization maintaining a malignant phenotype. Our previous studies showed that ANXA1 Knock-Out (KO) MIA PaCa-2 cells partially lost their migratory and invasive capabilities and also the metastatization process appeared affected in vivo. Here, we investigated the microRNA (miRNA) profile in ANXA1 KO cells finding that the modification in miRNA expression suggests the significant involvement of ANXA1 in PC development. In this study, we focused on miR-196a which appeared down modulated in absence of ANXA1. This miRNA is a well known oncogenic factor in several tumour models and it is able to trigger the agents of the epithelial to mesenchymal transition (EMT), like ANXA1. Our results show that the reintroduction in ANXA1 KO cells of miR-196a through the mimic sequence restored the early aggressive phenotype of MIA PaCa-2. Then, ANXA1 seems to support the expression of miR-196a and its role. On the other hand, this miRNA is able to mediate cytoskeletal dynamics and other protein functions promoting PC cell migration and invasion. This work describes the correlation between ANXA1 and specific miRNA sequences, particularly miR-196a. These results could lead to further information on ANXA1 intracellular role in PC, explaining other aspects that are apart from its tumorigenic behaviour.

## 1. Introduction

Annexin A1 (ANXA1) is a 37 kDa Ca^2+^-regulated phospholipid-binding protein that is involved in a wide range of physio-pathological processes, including cancer development [1,2,3]. In previous studies, the decreased expression of ANXA1 has been shown to be responsible of a strong delay of proliferation, migration/invasion, and angiogenesis on melanoma, Lewis lung carcinoma, non-small cell lung, breast, and prostate cancer models [4,5,6,7]. Thus, ANXA1 represents a possible target for novel therapies and/or a potential biomarker for cancer diagnosis and screening [8,9].

The oncogenic role of ANXA1 has been found also in pancreatic cancer (PC), where protein expression directly correlates with patients’ poor prognosis [10,11]. Recently, we have shown that ANXA1 enhances cell migration and invasion, acting both directly in the intracellular compartment and indirectly through the interaction with the formyl peptide receptors (FPRs) [12,13]. Moreover, the establishment of an ANXA1 knock-out (KO) in our in vitro model by CRISPR/Cas9 genome editing system on MIA PaCa-2 PC cells revealed the deregulation of a large number of proteins that are involved in cytoskeletal organization. This induced the reversion of epithelial to mesenchymal transition (EMT), leading to a less aggressive phenotype. Interestingly, we have also confirmed these finding in vivo, since the orthotopic xenografts of ANXA1 KO MIA PaCa-2 have shown a strong decrease of the metastatization rate, highlighting a severe oncogenic role of this protein in PC progression [13].

The microRNAs (miRNAs) are endogenous, non-coding single-stranded RNA molecules belonging to a family of key components in cells and participating in multiple biological processes mostly as negative gene regulators. Based on the results that were obtained in the last decade, some miRNAs are emerging as biomarkers for the detection, diagnosis, classification, and treatment of cancer [14,15,16,17]. Indeed, it has been shown that the dysregulation of miRNAs contribute to different human pathologies, including cancer [18,19,20]. Recently, the investigation of miRNA profile has been revealed useful as diagnostic screening method also for PC, where, among the most characterized miRNAs, miR-196a has been associated with recurrence and shorter survival [21,22]. There are three miR-196 genes in human cells: miR-196a-1, miR-196a-2, and miR-196b deriving from loci in the homeobox (HOX) gene clusters. In cancer, the first identified targets of miR-196a were HOXA and HOXB genes in primary adult acute myeloid leukaemia and HOXC8 and HOXB7 in melanoma [23,24,25]. miR-196a is considered to be a discriminating factor between PC and normal pancreas, since it is upregulated only in tumour samples [26,27]. Furthermore, it has been shown in vitro that miR-196a has an expression profile directly correlated with PC cell aggressiveness, as assessed by EMT [28,29].

Understanding the molecular mechanisms of ANXA1 in cancer also involves the investigation of its relationship with miRNAs. In fact, the protein can regulate downstream gene activation and transcription factors and, conversely, can be regulated by them. In this regulatory network, it reported a negative feedback loop between ANXA1 and miR-196a in systems as head and neck, endometrial, esophageal, and breast cancers [30,31]. In PC progression, ANXA1 and miR-196a have been independently studied as separated molecular markers of neoplastic transformation and poor prognosis. Overall, the reported information represents an appealing starting point for a crossed study in order to identify a possible relationship between them.

In this study, we performed a miRNA profiling analysis of ANXA1 KO MIA PaCa-2 cells as compared with PGS (empty vector, technical control) ones. We focused on miR-196a, which remarkably resulted among the down-modulated miRNAs in the absence of ANXA1. Furthermore, since both ANXA1 and miR-196a are able to trigger the mechanisms leading to the EMT, we have investigated some aspects of this process in vitro to describe how the miRNA could affect PC progression in absence of ANXA1.

## 2. Results

### 2.1. Differential miRNA Expression Profile in PGS and ANXA1 KO MIA PaCa-2 Cells

**Figure 1:**
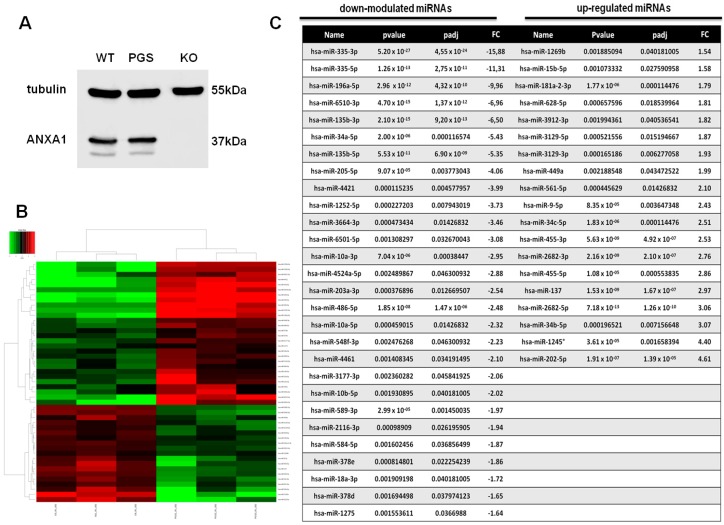
(**A**) Western blot of Annexin A1 (ANXA1) in wild type (WT), PGS MIA PaCa-2, and ANXA1 KO MIA PaCa-2 cells; (**B**) Heat map of the differential miRNA profile expression in PGS and ANXA1 KO MIA PaCa-2 cells. (padj ≤ 0.05, FC ≥ 1.5); and (**C**) Table listing all 19 upregulated and 28 downregulated miRNAs. The degree of differentially expressed miRNAs are expressed as *p* value, *p* value adjustment (padj) and the fold changes (FC).

The effects of genomic deletion of ANXA1 in PC cells have been previously evaluated using in vitro models of ANXA1 KO MIA PaCa-2 cells created using CRISPR/Cas9 genome editing system [13]. The Western blot confirming the lack of ANXA1 when compared with wild type (WT) and PGS MIA PaCa-2 cells is shown in Figure 1A. PGS cells have been obtained transfecting WT MIA PaCa-2 cells with an empty vector and have been used as our control, since they have a very similar behaviour if compared with the parental cell line [13]. Thus, we investigated differentially expressed ed miRNAs and conducted their target prediction with toolbox iSmaRT [32], as reported in Material and Methods section. Our analysis revealed that 47 miRNAs were differentially expressed after ANXA1 silencing. As reported in the heat map in Figure 1B, 19 miRNAs appeared to be upregulated and 28 downregulated. These miRNAs are listed in Figure 1C. The technical aspects of the performed analysis are reported in Supplementary Figure S1, which describes the length distribution of the revealed reads, the distance measure calculated among the expression profiles of each sample and the principal component analysis (PCA).

### 2.2. The miR-196a-5p Mimic Increased the Migration of PGS and ANXA1 KO MIA PaCa-2 Cells

**Figure 2:**
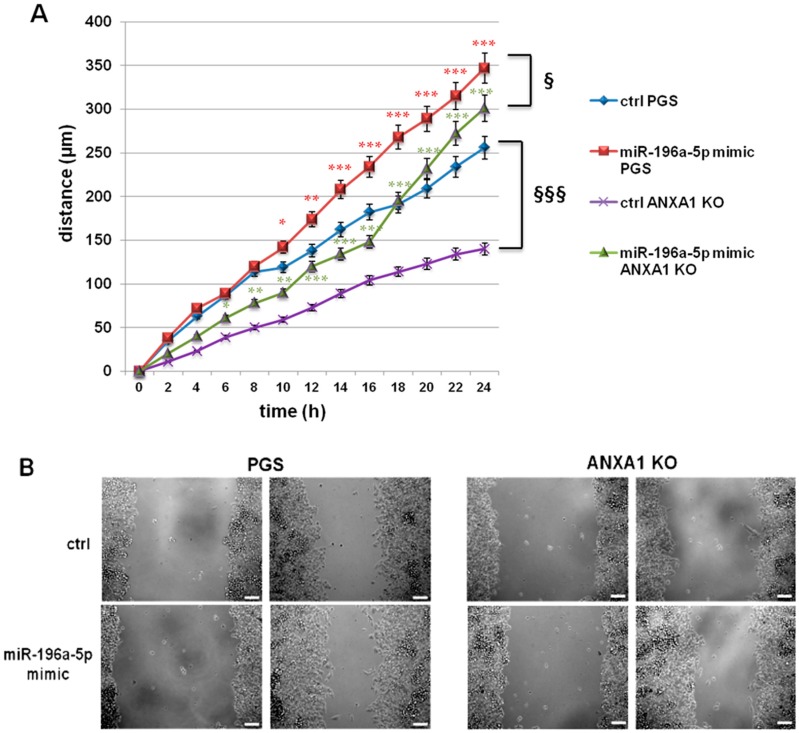
(**A**) Wound healing assay on PGS and ANXA1 KO MIA PaCa-2 cells; * *p* < 0.05, ** *p* < 0.01, *** *p* < 0.001 mimic treated vs. not treated cells. § *p* < 0.05, §§§ *p* < 0.001 ANXA1 KO vs. PGS MIA PaCa-2 cells. The migration rate was determined by measuring the distances covered by individual cells from the initial time to the selected time-points (bar of distance tool, Leica ASF software). The data are representative of three independent experiments ± SEM; and (**B**) Representative images that were captured by TIME LAPSE microscope of PGS and ANXA1 KO MIA PaCa-2 at 0 and 24 h from produced wounds. Magnification 10×. Bar: 100 µm.

Several studies have investigated the role of miRNAs in PC. Many of them focused on miR-196a as a potential marker since it appears to be involved in the acquisition of aggressiveness and correlated to poor prognosis [26,28,33]. When considering the significant down-modulation of mR-196a-5p in ANXA1 KO MIA PaCa-2 cells, we transfected PGS and ANXA1 KO cells with the relative mimic in order to highlight its role in our system. The transfection efficiency has been tested in Supplementary Figure S2, where the cell counts taken, as reported in Material and Methods section are shown. Initially, we performed a Wound healing assay to test cell migratory capability. In Figure 2A, a graphical representation of the increase of migration rate both in PGS and in ANXA1 KO MIA PaCa-2 cells is shown. This increase appears more evident in ANXA1 KO cells since this clone confirmed to be characterized by a lower migratory behaviour [13]. These results are supported by representative images (Figure 2B).

### 2.3. miR-196a-5p Affected PGS and ANXA1 KO MIA PaCa-2 Invasive Behaviour

**Figure 3:**
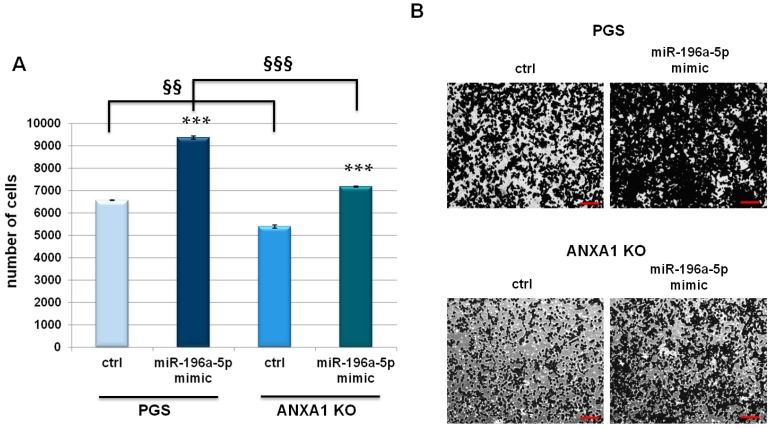
(**A**) Invasion assay of ANXA1 KO and PGS MIA PaCa-2 cells. Data represent mean cell counts of 12 separate fields per well ± SEM of three experiments. *** *p* < 0.001 mimic treated vs. not treated cells. §§ *p* < 0.01, §§§ *p* < 0.001 ANXA1 KO vs. PGS MIA PaCa-2 cells; and (**B**) Representative images of analysed fields of invasion assay. Magnification 10×. Bar = 150 μm.

Following the same transfection procedures, an invasion assay through the coating of matrigel with PGS and ANXA1 KO MIA PaCa-2 cells was performed. In presence of miR-196a-5p mimic a strong increase of invasion rate of the analyzed clones was observed (Figure 3A). Figure 3B show the representative images resulting from cell invasion.

### 2.4. The miR-196a-5p Mimic Induced the Increase of Some EMT Markers in PGS and ANXA1 KO MIA PaCa-2 Cells

**Figure 4:**
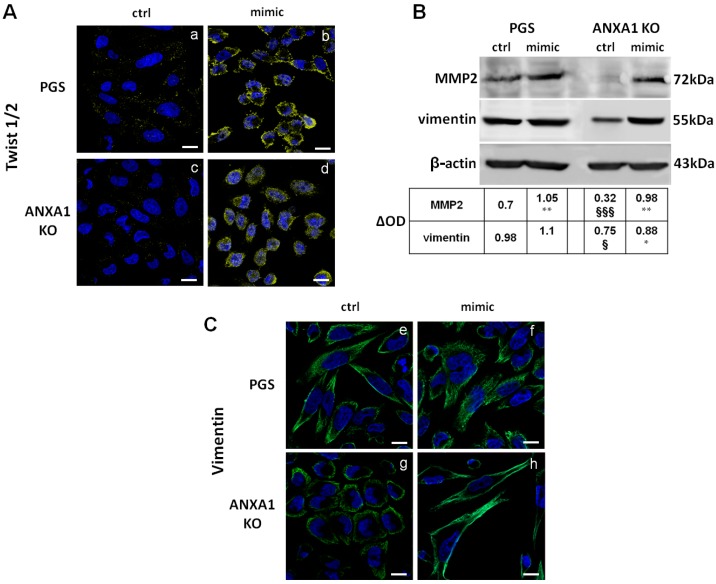
(**A**) Immunofluorescence analysis to detect Twist 1/2 (yellow) activation following the effects of miR-196a-5p mimic of PGS (panels (**a**) and (**b**)) and ANXA1 KO cells (panels (**c**) and (**d**)). Nuclei were stained in blue with Hoechst 33342. Magnification 63 × 1.4 NA. Bar = 10 µm; (**B**) Western blot of MMP2 and vimentin expression in PGS and ANXA1 KO MIA PaCa-2 cells in presence or not of miR-196a-5p mimic. All protein levels are normalized on β-actin levels. For densitometry analysis, the blots were exposed to Las4000 (GE Healthcare Life Sciences, Buckinghamshire, UK) and the relative intensities of bands were determined using ImageQuant software (GE Healthcare Life Sciences, Buckinghamshire, UK). * *p* < 0.05, ** *p* < 0.01 mimic treated vs. not treated cells. § *p* < 0.05, §§§ *p* < 0.001 ANXA1 KO vs. PGS MIA PaCa-2 cells; and (**C**) Immunofluorescence for vimentin (green) on PGS (panels (**e**) and (**f**)) and ANXA1 KO cells (panels (**g**) and (**h**)) treated or not with mimic. Magnification 63 × 1.4 NA. Bar = 10 µm. Cells have been harvested after 48 h of transfection. Data are representative of three experiments with similar results.

miRNAs have a significant role in the EMT process, through regulation of key genes, such as ZEB1, ZEB2, Snail, Twist [34]. Based on the knowledge that EMT is significantly associated with metastatization, we studied the main markers that are involved in this transformation. In Figure 4A, the increased expression of Twist 1/2 and its activation are shown. Indeed, this factor appeared to translocate from cytosol to the nucleus after 48 h of transfection with miR-196a-5p mimic, both in PGS (panels a and b) and in ANXA1 KO (panels c and d) MIA PaCa-2 cells. Based on the increase of migration and invasion processes in the presence of miRNA, we also highlighted the expression of matrix metalloproteinases (MMP) 2 [35]. An increased expression of this protein in presence of miR-196a-5p mimic was found. We have been also able to prove the significant differences between the MMP2 basal levels of PGS and ANXA1 KO MIA PaCa-2 cells, confirming their different invasion ability (Figure 4B) [13]. Western blot in Figure 4B also shows the increase of vimentin expression induced by mimic and its different levels in PGS and ANXA1 KO MIA PaCa-2 cells. These results are further proved by immunofluorescence assay in Figure 4C (panels e and f for PGS; g and h for ANXA1 KO). Particularly, panel h shows the strong morphological change of ANXA1 KO cells, which appears as fibroblast-shaped when being transfected with miR-196a-5p mimic. In Figure S3, we showed the quantitative analysis of protein levels through immunofluorescence assays.

### 2.5. The miR-196a-5p Mimic Led to Cytoskeletal Organization Particularly on ANXA1 KO MIA PaCa-2 Cells

**Figure 5:**
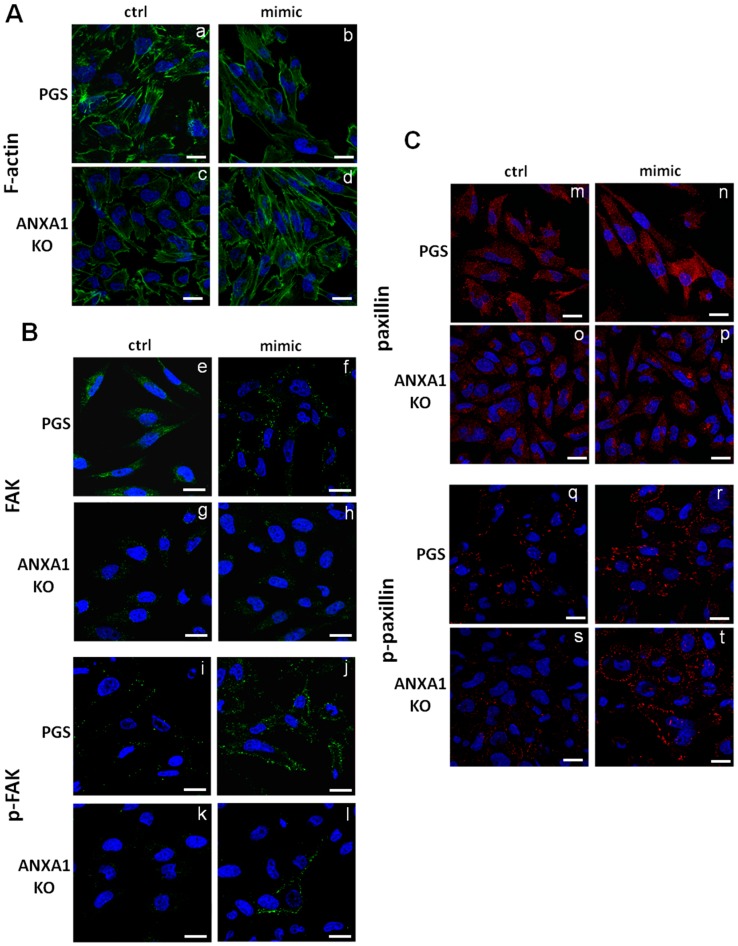
Immunofluorescence analysis. (**A**) F-actin organization (green) in PGS (panels (**a**) and (**b**)) and ANXA1 (panels (**c**) and (**d**)); (**B**) focal adhesion kinases (FAK) (PGS, panels (**e**) and (**f**); ANXA1 KO panels (**g**) and (**h**)) and phospho-FAK (PGS, panels (**i**) and (**j**); ANXA1 KO panels (**k**) and (**l**)) (FAK and phospho-FAK are shown in green); and (**C**) paxillin (PGS, panels (**m**) and (**n**); ANXA1 KO panels (**o**) and (**p**)) and phospho-paxillin (PGS, panels (**q**) and (**r**); ANXA1 KO panels (**s**) and (**t**)) treated or not with miR-196a-5p mimic (paxillin and phospho-paxillin are shown in green). Nuclei were stained in blue with Hoechst 33342. Magnification 63 × 1.4 NA. Bar = 10 µm. The data relative to cells harvested after 48 h from the transfection, are representative of 3 experiments with similar results.

Next, we performed immunofluorescence assays in order to analyze the cytoskeletal organization exploring F-actin polymerization by phalloidin staining (Figure 5A, panels a and b for PGS; panels c and d for ANXA1 KO). The actin cytoskeleton appeared reorganized in ANXA1 KO cells in the presence of miR-196a-5p mimic. Particularly, the signal of F-actin fibers seem to be focused at the leading edges. This difference appears more significant if compared with the basal condition of ANXA1 KO cells that do not have strong migratory abilities [13]. Furthermore, we focused on the regulation of focal adhesion kinases (FAK), which are involved in the promotion of cell migration. These kinases induce several downstream signalling pathways when activated by phosphorylation [36]. The transfection with miR-196a-5p mimic did not induce a significant change of FAK expression at the plasma membrane (Figure 5B, panels e and f for PGS and panels g and h for ANXA1 KO). In contrast, a notable increase of the phosphorylated form of FAK (p-FAK) in PGS and even more in ANXA1 KO MIA PaCa-2 cells after 48 h of transfection was observed (Figure 5B, panels i and j for PGS and panels k and l for ANXA1 KO). Additionally, the paxillin expression and activation was investigated, since this protein represents a scaffold for recruitment of many signalling proteins to the plasma membrane, including FAK [37]. Paxillin expression and translocation to the plasma membrane did not notably change (Figure 5C, panels m and n for PGS and o and p for ANXA1 KO). Differently, p-paxillin, the protein activated form, appeared strongly expressed at the plasma membrane in the presence of miR-196a-5p mimic. PGS MIA PaCa-2 cells showed a lightly less evident increase of p-paxillin signal if compared with ANXA1 KO ones at the same conditions (Figure 5C, panels q and r for PGS and panels s and t for ANXA1 KO). In Figure S3, the quantitative analysis of protein levels through immunofluorescence assays is shown.

## 3. Discussion

In this study, the miRNA regulation profile associated to the ANXA1 expression in human PC MIA PaCa-2 cells is reported. Interestingly, we observed the differential expression for 47 miRNAs revealed in ANXA1 KO obtained by CRISPR/Cas9 genome editing in vitro system. The analysis of these miRNAs disclosed further remarkable information about the involvement of ANXA1 in PC progression. Indeed, regarding the down-modulated sequences in ANXA1 KO cells, we recognized miR-196a, miR-205, miR-10a, and miR-10b, which are known as oncogenic factor, inducing proliferation, migration, and invasion in several tumor models [18,38,39,40,41,42,43,44,45]. On the other hand, miR-34c, miR-455, miR-202, miR-137, which are upregulated in absence of ANXA1, exert cancer suppression [46,47,48,49,50,51,52].

Overall, these profound modifications are in accordance with our previous findings, which showed the dysregulation of a large number of proteins in ANXA1 KO cells [13]. Indeed, the analysis of the proteomic profile has reported many changes concerned the cytoskeletal dynamics and affected the migratory and invasive ability of MIA PaCa-2. These cells became less aggressive when lacking ANXA1 and were prone to the reversion of the EMT, as further shown by orthotopic xenografts of ANXA1 KO MIA PaCa-2 cells, where the absence of this protein negatively affected the prominent liver metastatization [13].

Despite that miR-196a is not the more dysregulated miRNAs among the listed ones, we selected this sequence since it has been found playing a critical role in the pathogenesis of several cancer models [53,54,55,56,57], including the pancreatic one. Interestingly, in this system, it can be used as the discriminating factor of cancer lesions from normal tissues and chronic pancreatitis [26,27,28]. Previous reports about the putative correlation ANXA1/miR-196a described an inverse relationship between them [30,58,59,60]. These experimental evidences led us to first investigate the functions of this miRNA sequences correlated with ANXA1. Furthermore, we have been intrigued by the lack of detailed information about the correlation of miR-196a and ANXA1 in PC, while the miRNA-mediated mechanism in the reduced expression of ANXA1 in breast, endometrial, and esophageal cancers has been explained [30].

Taken together, this knowledge encouraged us to focus on the down-modulation of miR196a in ANXA1 KO MIA PaCa-2 cells, despite that this kind of correlation appeared inverse in numerous tumour models. Furthermore, both ANXA1 and miR-196a are able to enhance the metastatization process in PC. Since ANXA1 and miR-196a are involved in the induction of a mesenchymal as well as a more aggressive phenotype [13,27], we evaluated cell migration and invasion showing the increase of the rate of these processes in presence of miR-196a-5p mimic. Thus, we suggest that the aberrant expression of miRNA, as well as ANXA1, promotes PC progression.

The activation of Twist 1/2, as a well characterized transcription factor activating the EMT, Lamouille et al. [34] proves the involvement of miR-196a in the acquisition of a more aggressive phenotype and it represents the first experimental evidence of a relationship between Twist 1/2 and miR-196a. The increase of other EMT markers as vimentin and MMP-2 also helped us to validate the strong morphological change of MIA PaCa-2 cells in the presence of miR-196a-5p mimic. Interestingly, the acquisition of a marked fibroblast-like shape appeared to be particularly evident in ANXA1 KO cells. About these characteristics, the modulation of cytoskeletal organization still represents the keystone of our study not only in term of F-actin rearrangement, but particularly for the cell adhesion/motility complex. Moreover, the F-actin network adjacent to the plasma membrane at the level of the leading edges of cells is known to be involved in lamellipodia extension. Therefore, we focused on the on focal adhesion formation, since it is not reported the involvement of miR-196a, differently from ANXA1 which is well characterized in this pattern [12,61,62].

The findings obtained about FAK/p-FAK and paxillin/p-paxillin suggest that the absence of ANXA1 and the consecutive down-modulation of miR-196a-5p in MIA PaCa-2 cells lead to the acquisition of a less aggressive behaviour. FAKs represent a crucial cross-talking factor in cell motility, associating the cytoplasmic domains of integrins to the actin filaments [63]. On the other hand, paxillin is able to bind several proteins that contribute to the organization of the cytoskeleton [37]. Thus, these proteins demonstrate an additional protrusive function in the formation of new lamellipodia via the Rac-induced polymerization of actin [64]. It is well known that the altered expression levels and the phosphorylation statuses of FAK and paxillin are crucial in metastasis and angiogenesis pathways.

In the presence of miR-196a-5p mimic this mesenchymal phenotype was enhanced more in ANXA1 KO MIA PaCa-2 cells than in PGS ones. Generally, we expected these differences since ANXA1 KO cells present epithelial features and the induction of EMT would be more evident when compared to PGS MIA PaCa-2.

Altogether, our data show two main aspects explicating the role of miR-196a as a function of ANXA1 in MIA PaCa-2 cells. First, the reintroduction of miR-196a was able to enhance the mesenchymal features, restoring the early phenotype in ANXA1 KO MIA PaCa-2 cells. Next, we could speculate that ANXA1 support the expression of miR-196a and its oncogenic role during the PC progression and that some of ANXA1 functions could be mediated also by this miRNA. This work might describe a new pattern about the involvement of ANXA1 and miRNAs in PC invasiveness, and lead to a better understanding of the protein intracellular role. The mechanistic details of the direct or indirect correlation ANXA1/miR-196a will be the aim of future studies.

## 4. Material and Methods

### 4.1. Cell Cultures

MIA PaCa-2 cells are immortalized epithelial cells of human pancreatic carcinoma. They were purchased from ATCC (ATCC CRL-1420, Manassas, VA, USA) and cultured in high glucose DMEM containing L-Glutamine 2 mM, 10% heat-inactivated fetal bovine serum (FBS), 2.5% heat inactivated horse serum (HS), and 10,000 U/mL penicillin and 10 mg/mL streptomycin (Euroclone, Milan, Italy). Cells were stained at 37 °C in 5% CO_2_-95% air humidified atmosphere. PGS and ANXA1 KO MIA PaCa-2 cells were obtained using CRISPR-Cas9 plasmid that was purchased from GenScript (Piscataway Township, NJ, USA), as reported in [13]. These clones were kept in selection by 700 μg/mL neomycin (Euroclone, Milan, Italy).

### 4.2. RNA Isolation and Quality Controls

Total RNA was extracted from PGS and ANXA1 KO MIA PaCa-2 cells using TRI Reagent^®^ (Sigma-Aldrich, St. Louis, MO, USA). RNA concentration was assayed with a NanoDrop 2000c spectrophotometer (Thermo Fisher Scientific, Waltham, MA, USA) and its quality assessed with the Agilent 4200 TapeStation with Agilent RNA ScreenTape Assay (Agilent Technologies, Santa Clara, CA, USA).

### 4.3. Small RNA Sequencing and Data Analysis

cDNA libraries were prepared with 1 µg of starting total RNA and using TruSeq Small RNA Sample Preparation kit, according to TruSeq protocol (Illumina, San Diego, CA, USA). Sequencing libraries were subjected to quality controls by Agilent 2100 Bioanalyzer using Agilent DNA High Sensitivity kit (Agilent Technologies, Santa Clara, CA, USA) and quantified with Qubit^®^ 2.0 Fluorometer (Invitrogen Co., Carlsbad, CA, USA). Then, individual libraries with unique indices were pooled at equimolar 4 nM final concentration, including the Phix Control Library. The final pool was sequenced to a final concentration of 1.8 pmol on Illumina NextSeq platform while using High Output V2 Kit. Small RNA sequencing data was analyzed using iSmaRT [32] to identify the microRNA (miRBase v21, genome assembly GRCh37/hg19) with Minimum Read Count of 3. To recognize differentially expressed miRNAs between ANXA1 KO MIA PaCa-2 cells versus PGS samples, DESeq2 algorithm was used [65] with FDR ≤ 0.05 and Fold-Change ≥ |1.5|. Data integration, heatmap visualization of differentially expressed transcripts, and functional enrichment plots were done with R/Bioconductor packages and the Multi Experiment Viewer software (MeV v4.9, Sun Microsystems, Santa Clara, CA, USA) [66]. Raw data is available on ArrayExpress with accession number E-MTAB-6599.

### 4.4. Mimic Transfection

PGS, ANXA1 KO MIA PaCa-2 cells were transfected with hsa-miR196a-5p UAGGUAGUUUCAUGUUGUUGGG mimic and used at a final concentration of 100 nM for 24 and 48 h. miRIDIAN microRNA Mimic Negative Control #1 CN-001000-01-05 has been used as negative control. The sequences were purchased from Dharmacon (Lafayette, CO, USA) transfected using Lipofectamine 2000 Reagent (Life technologies Corporation, Carlsbad, CA, USA), according to the manufacturer’s instructions. In order to test the transfection efficiency, for each experiment with mimic, we performed a control co-transfecting the sequence of our interest with Tye 563 probe 1 nM (Integrated DNA Technologies, Coralville, IA, USA). After 24 and 48 h from the transfection, images of fluorescence signal have been taken at Integrated Live Cell Workstation Leica AF-6000 LX.

### 4.5. In Vitro Wound-Healing

A wound was produced on the confluent monolayer of cells by scraping them with a pipette tip. The experimental points have been PGS and ANXA1 KO MIA PaCa-2 transfected or not with miR-196a-5p mimic for 24 h and further treated with mitomycin C (10 μg/mL, Sigma-Aldrich, St. Louis, MO, USA) in order to ensure the block of mitosis. The wounded cells were incubated at 37 °C in a humidified and equilibrated (5% *v*/*v* CO_2_) incubation chamber of an Integrated Live Cell Workstation Leica AF-6000 LX. A 10× phase contrast objective was used to record cell movements with a frequency of acquisition of 10 min. The migration rate of individual cells was determined by measuring the distances that were covered from the initial time to the selected time-points (at 24 h) (bar of distance tool, Leica ASF software). Three independent experiments were performed. For each wound, five different positions were registered, and for each position, ten different cells were randomly selected on both side of the scratch to measure the migration distances.

### 4.6. Invasion Assay

Cell invasiveness was studied using the Trans-well Cell Culture (12 mm diameter, 8.0-fim pore size) purchased form Corning Incorporated (New York, NY, USA), as previously described [12]. Briefly, 9 × 10^4^ PGS, ANXA1 KO MIA PaCa-2 cells, transfected or not with miR-196a-5p mimic 24 h before, were seeded on the matrigel coating and maintained at 37 °C in 5% CO_2_-95% air humidified atmosphere. After 24 h, the Trans-well Cell Culture chambers were washed twice with PBS and fixed with 4% p-formaldehyde for 10 min, and then with 100% methanol for 20 min. Later, the fixed cells were stained with crystal violet (0.5% *w*/*v* in a *v*/*v* solution of 20% methanol/distilled water; Merck Chemicals, Darmstadt, Germany) for 15 min. Next, the chambers were washed again in PBS and cleaned with a cotton bud to remove crystal violet exceedance. All of the experimental points were treated with mitomycin C (10 μg/mL, Sigma-Aldrich, St. Louis, MO, USA) to ensure the block of mitosis. The number of cells that had migrated to the lower surface was counted in twelve random fields using EVOS light microscope (10×) (Life technologies Corporation, Carlsbad, CA, USA).

### 4.7. Confocal Microscopy

PGS and ANXA1 KO MIA PaCa-2 cells, which were fixed in p-formaldehyde (4% *v*/*v* in PBS; Lonza, Basel, Switzerland), were permeabilized with Triton X-100 (0.4% *v*/*v* in PBS; Lonza, Basilea, Switzerland), blocked with goat serum (20% *v*/*v* in PBS; Lonza, Basilea, Switzerland) and then incubated with anti- vimentin (mouse monoclonal, 1:500; Santa Cruz Biotechnologies, Dallas, TX, USA), Twist 1/2 (rabbit polyclonal, 1:100; GeneTex, Irvine, CA, USA), FAK (mouse monoclonal; 1:100; BD Transduction Laboratories, Franklin Lakes, NJ, USA), p-FAK (mouse monoclonal; 1:100; Cell Signaling Technology, Danvers, MA, USA), paxillin (rabbit polyclonal, 1:100; Cell Signaling Technology), p-paxillin (rabbit polyclonal, 1:100; Cell Signaling Technology) overnight at 4 °C. After two washing steps, the cells were incubated with AlexaFluor anti-mouse 488 and anti-rabbit 555 (1:500; Molecular Probes, Eugene, OR, USA) for 2 h at room temperature (RT), or with FITC-conjugated anti-F-actin (5 µg/mL; Phalloidin-FITC, Sigma-Aldrich, St. Louis, MO, USA) for 30 min at RT in the dark. To detect nucleus, samples were incubated with Hoechst 33342 (1:1000; Molecular Probes, Eugene, OR, USA) and excited with a 458 nm Ar laser. A 488 nm Ar or a 555 nm He-Ne laser was used to detect emission signals from target stains. Samples were vertically scanned from the bottom of the coverslip with a total depth of 5 μm and a 63× (1.40 NA) Plan-Apochromat oil-immersion objective. Images and scale bars were generated with Zeiss ZEN Confocal Software (Carl Zeiss MicroImaging GmbH, Jena, Germany) and presented as single stack. Images were processed using ImageJ software (NIH, Bethesda, MD, USA), Adobe Photoshop CS version 5.0, and figures were assembled using Microsoft PowerPoint (Microsoft Corporation, Redmond, WA, USA). For immunofluorescence analysis and quantification, the final images were generated using Adobe Photoshop CS4, version 11.0. Quantifications were performed from multichannel images obtained using a 63× objective using ImageJ, marking either the cell perimeter or the nucleus as the region of interest and calculating integrated densities per area from the appropriate channel. A minimum of 50 cells were analyzed for each data set. The obtained mean value was used to compare experimental groups.

### 4.8. Western Blotting

Protein expression was examined by Western blot, as previously described [12]. Proteins were visualized using the chemioluminescence detection system (Amersham biosciences, Buckinghamshire, UK) after incubation with primary antibodies against rabbit polyclonal ANXA1 (1:10,000; Invitrogen, Carlsbad, CA, USA), MMP2 (1:1000; GeneTex, Irvine, CA, USA), and mouse monoclonal vimentin (1:1000; Santa Cruz Biotechnologies, Dallas, TX, USA), α-tubulin (1:1000; Sigma-Aldrich, St. Louis, MO, USA), and β-actin (1:1000; Sigma-Aldrich, St. Louis, MO, USA). The blots were exposed to Las4000 (GE Healthcare Life Sciences, Buckinghamshire, UK) and the relative band intensities were determined using ImageQuant software (GE Healthcare Life Sciences, Buckinghamshire, UK). Results were considered to be significant if *p* < 0.01.

### 4.9. Statistical Analysis

Data analyses and statistical evaluations were carried out using Microsoft Excel; the number of independent experiments, standard deviations/errors, and *p*-values are indicated in the figure legends. All of the results are the mean ± standard error of measurement (SEM) of at least three experiments performed in triplicate. Statistical comparisons between groups were made using two-tailed *t*-test comparing two variables. Differences were considered to be significant if *p* < 0.05, *p* < 0.01 and *p* < 0.001.

## 5. Conclusions

The knowledge of a correlation between ANXA1 and specific miRNA sequences, such as miR-196a, adds an important element in the combinatory panel of specific factors and encourages future investigations. Indeed, in PC both the protein and miR-196a represent important factors promoting metastatization. However, the integration of ANXA1 in a more complex panel of biomarkers for PC screening and the differential diagnosis remains our central intent. Future studies will address the relationship between ANXA1 and other miRNAs, either upregulated or downregulated, founded in our analyses in order to verify a possible tight connection between these agents. The results may support the use of ANXA1 as prognostic and/or clinical tool for PC.

## Supplementary Materials

The following are available online at http://www.mdpi.com/1422-0067/19/7/1967/s1. Accompanies this paper. Figure S1: (A) Length distribution of reads in a representative small RNA sequencing library; (B) Hierarchical clustering on normalized expression count on identified miRNAs for each sample; and (C) Principal Component Analysis on normalized expression count of identified miRNAs for each sample; Figure S2: (A) Representative images generated with Leica ASF software of 1 nM tye 563 fluorescence signal merged on bright field at 48 h from co-transfection with miR-196a-5p mimic 100 nM on PGS and ANXA1 KO MIA PaCa-2 cells. Magnification 10×. Bar = 150 μm (panels (a) and (c)). Magnification 20×. Bar = 300 μm (panels (b) and (d)); and (B) Cell count calculated as percentage of red fluorescent signal on total number of cells and calculated by ImageJ software. The result represents a mean value of ten fields randomly selected and registered for each experimental condition. The percentages of cells showing red signal inside are 66.4% for PGS cells and 64.2% for ANXA1 KO. Figure S3: Quantification of immunofluorescence shown in Figure 4: (A) Twist 1/2 ((a)–(d)) and (B) vimentin ((e)–(h)), and in Figure 5: (C) F-actin ((a)–(d)), (D) FAK/p-FAK ((e)–(l)), (E) paxillin/p-paxillin ((m)–(t)) as percent of fluorescence intensity per cell area. ** *p* < 0.01, *** *p* < 0.001 mimic treated vs. not treated cells. §§ *p* < 0.01 ANXA1 KO vs. PGS MIA PaCa-2 cells.
