# Supplementary figures and images for: miR-196a Is Able to Restore the Aggressive Phenotype of Annexin A1 Knock-Out in Pancreatic Cancer Cells by CRISPR/Cas9 Genome Editing
Source: Int J Mol Sci. 2018 Jul 6;19(7):1967. doi: 10.3390/ijms19071967 (PMC6073506; doi:10.3390/ijms19071967)

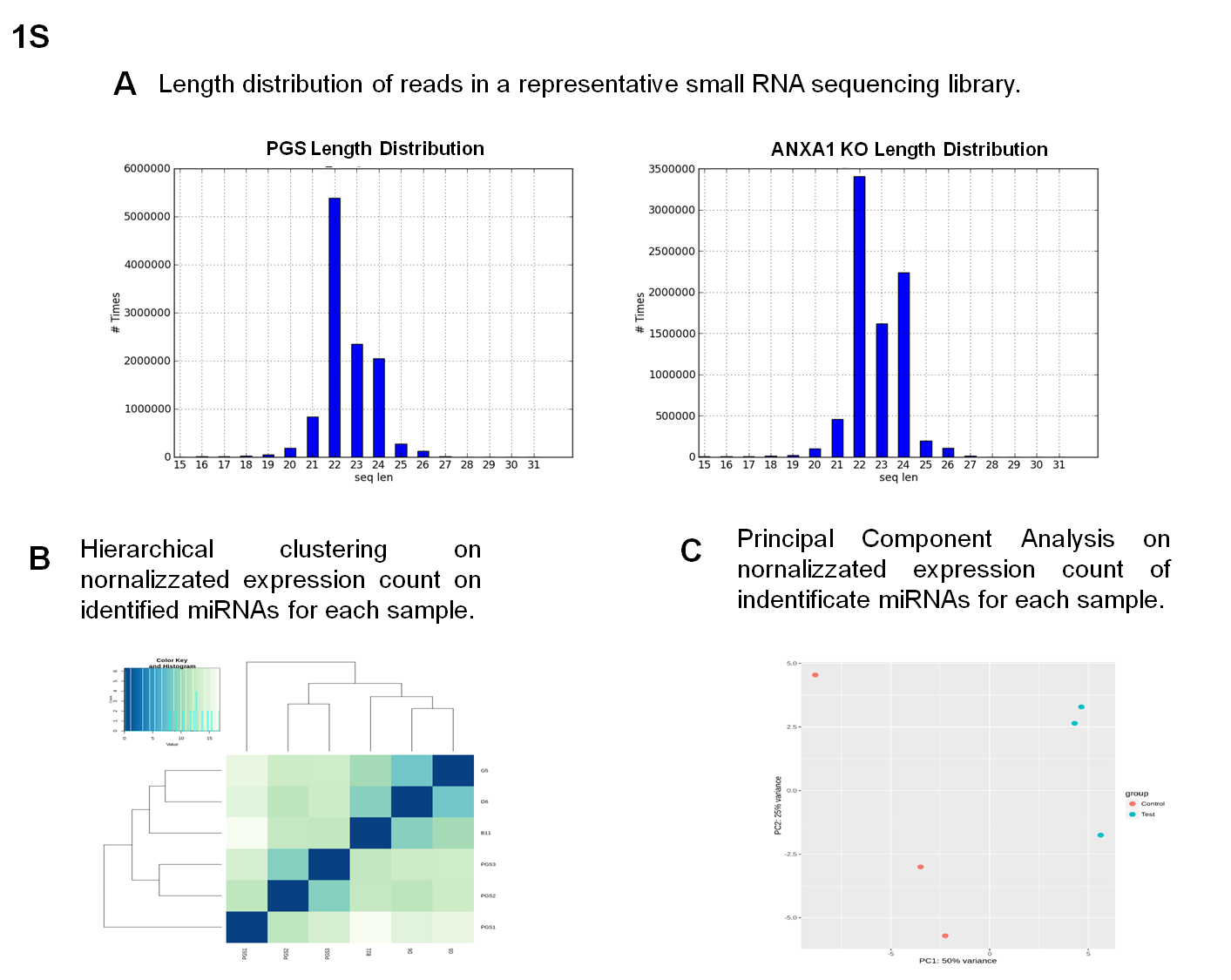

Supplement: Supplementary file 1 [file ijms-19-01967-s001.zip › Supplementary figure 1.tif]

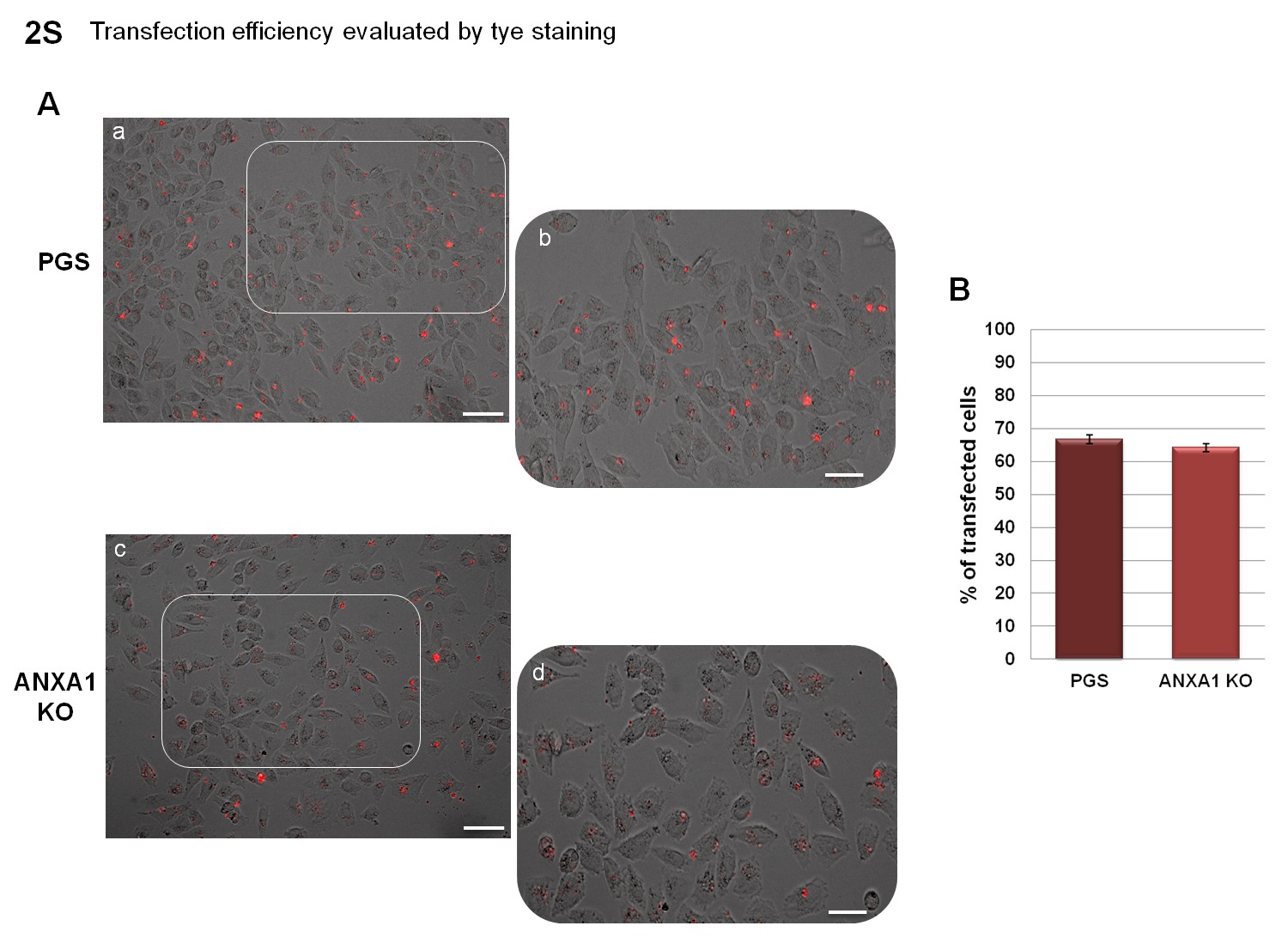

Supplement: Supplementary file 1 [file ijms-19-01967-s001.zip › Supplementary figure 2.tif]

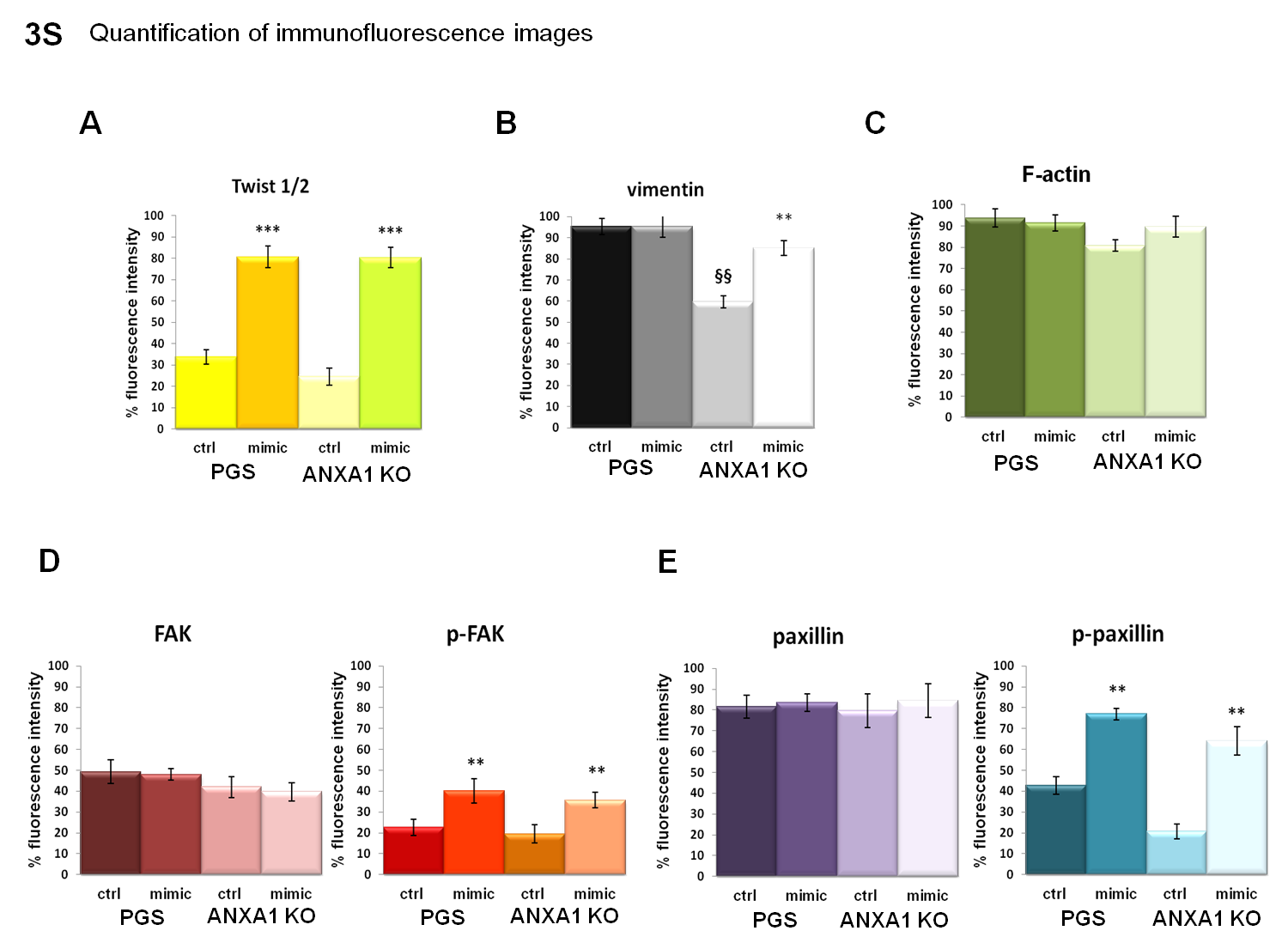

Supplement: Supplementary file 1 [file ijms-19-01967-s001.zip › Supplementary figure 3.tif]
